# Genomics-Based Insights Into the Biosynthesis and Unusually High Accumulation of Free Fatty Acids by *Streptomyces* sp. NP10

**DOI:** 10.3389/fmicb.2018.01302

**Published:** 2018-06-19

**Authors:** Olha Schneider, Tatjana Ilic-Tomic, Christian Rückert, Jörn Kalinowski, Marija S. Genčić, Milena Z. Živković, Nada Stankovic, Niko S. Radulović, Branka Vasiljevic, Jasmina Nikodinovic-Runic, Sergey B. Zotchev

**Affiliations:** ^1^Department of Biotechnology, Norwegian University of Science and Technology, Trondheim, Norway; ^2^Institute of Molecular Genetics and Genetic Engineering, University of Belgrade, Belgrade, Serbia; ^3^Center for Biotechnology, Bielefeld University, Bielefeld, Germany; ^4^Department of Chemistry, Faculty of Science and Mathematics, University of Niš, Niš, Serbia; ^5^Department of Pharmacognosy, University of Vienna, Vienna, Austria

**Keywords:** *Streptomyces*, fatty acid biosynthesis, genome sequence, biosynthetic gene clusters, insertional inactivation

## Abstract

*Streptomyces* sp. NP10 was previously shown to synthesize large amounts of free fatty acids (FFAs). In this work, we report the first insights into the biosynthesis of these fatty acids (FAs) gained after genome sequencing and identification of the genes involved. Analysis of the *Streptomyces* sp. NP10 draft genome revealed that it is closely related to several strains of *Streptomyces griseus*. Comparative analyses of secondary metabolite biosynthetic gene clusters, as well as those presumably involved in FA biosynthesis, allowed identification of an unusual cluster C12-2, which could be identified in only one other *S. griseus*-related streptomycete. To prove the involvement of identified cluster in FFA biosynthesis, one of its three ketosynthase genes was insertionally inactivated to generate mutant strain mNP10. Accumulation of FFAs in mNP10 was almost completely abolished, reaching less than 0.01% compared to the wild-type strain. Cloning and transfer of the C12-2 cluster to the mNP10 mutant partially restored FFA production, albeit to a low level. The discovery of this rare FFA biosynthesis cluster opens possibilities for detailed characterization of the roles of individual genes and their products in the biosynthesis of FFAs in NP10.

## Introduction

Soil dwelling bacteria of the genus *Streptomyces* are well known for their ability to produce chemically diverse secondary metabolites (SMs). This genus, owing to its remarkable biosynthetic potential, is solely is responsible for originating about half of the antibiotics in today’s clinical use, not mentioning other bioactive molecules – anticancer agents, antifungals, biocontrol agents, and immunosuppressors (Chater, 2016). These compounds are SMs which, unlike components of the primary metabolism, such as amino acids, nucleotides, lipids, and carbohydrates, are not essential for the growth of *Streptomyces* bacteria. Nevertheless, SMs appear to be important for environmental adaptation, ensuring survival, and proliferation of the producing organism, for instance by inhibiting other microorganisms competing for nutritional sources (van der Meij et al., 2017). Recent advances in the genomics of *Streptomyces* bacteria, coupled with the development of bioinformatics tools revealed their enormous potential to biosynthesize various SMs, some of which have never been detected during cultivation in the laboratory (Baltz, 2017). New approaches are being developed for “genome mining," aimed at unlocking the hidden potential of these bacteria to produce SMs, which may be developed into new therapeutics (Kealey et al., 2017). At the same time, the ability of streptomycetes to produce compounds of interest other than those relevant for medicine, e.g., fatty acids (FAs), does not receive much attention. This is a clear shortcoming, since bacterial production of FA-based fuels (e.g., biodiesel) and commodity chemicals commonly produced from oil receives much attention (Handke et al., 2011). Although the pathway for the biosynthesis of common FAs in *Streptomyces* is rather well understood (Gago et al., 2011), details on the biosynthetic pathways for complex branched FAs are still missing.

Streptomycetes are known to synthesize saturated FAs using the FA synthase II complex consisting of FabD (malonyl-CoA:ACP transacylase), FabH (beta-ketoacyl-acyl carrier protein synthase III), AcpP (acyl carrier protein), and FabF (beta-ketoacyl-acyl carrier protein synthase II) (Revill et al., 2001). FabA, a 3-hydroxyacyl-ACP dehydratase, is likely to be part of the complex as well, catalyzing the dehydration step of chain elongation (Singh and Reynolds, 2016). FA profiles in *Streptomyces* are most likely determined by FabH protein, which has a broad substrate specificity for short-chain acyl CoA substrates, including branched starters such as isobutyryl, isovaleryl, and anti-isovaleryl CoAs. Thus, FabH can give rise to odd- and even-numbered FAs with a methyl branch at the ω-terminus, which represent the major component in the FA profile (Han et al., 1998). Nothing is known, however, on the biosynthesis of complex branched FAs, some of which have antibacterial activity (Zheng et al., 2010).

*Streptomyces* sp. NP10 (ISS613), an environmental isolate was previously shown to produce and excrete into the growth medium large amounts of free long-chain FAs (Ilic-Tomic et al., 2015). A large structural diversity of free fatty acids (FFAs) with over 50 different *n*- and branched-chain, (un)saturated, and cyclopropane FAs (C7–C30) has been demonstrated. There is a strong rationale behind elucidating mechanisms for overproduction of FFA and their export to medium, since it can be exploited in microbial biodiesel production. In the current study, we obtained draft genome sequence of *Streptomyces* sp. NP10 and analyzed it for genes that may potentially be involved in the biosynthesis of FAs and SMs. Using comparative genomics, a novel biosynthetic gene cluster (BGC) for FFA biosynthesis was identified and its functionality confirmed by gene disruption and complementation experiments. This work sets stage for a comprehensive investigation into the biosynthesis of unusual FFAs and determination of the roles of individual genes in this process.

## Materials and Methods

### *Streptomyces* sp. NP10 Genome Sequencing, Annotation, and Analysis

Strain *Streptomyces* sp. NP10 was deposited at the Institute of Soil Science (Belgrade, Serbia) culture collection ISS WDCM375 under accession number ISS613 and the 16S rRNA gene sequence was deposited in GenBank under accession number JQ288108 (Ilic-Tomic et al., 2015). For sequencing, 1 μg of gDNA was used to create a WGS shotgun library using the TruSeq PCR-free library preparation kit (Illumina). The library was sequenced on a MiSeq sequencing platform using the 600 cycle sequencing kit to obtain 2× 300 bp paired end reads. After sequencing, the reads were assembled using the Newbler *de novo* assembler v.2.8 (454, Roche). Afterwards, the automatic assembly was inspected using CONSED (Gordon and Green, 2013), resulting in a final, high quality draft genome of 7,677,865 bp in 42 contigs at 73.5-fold coverage. The contigs were automatically annotated using the PROKKA pipeline (Seemann, 2014), which resulted in the identification of 6,851 CDS, 76 tRNAs, and 3 ncRNAs. The rDNA operon was found split over three contigs, indicating substantial variability in the 16S–23S intergenic region as well as in a region within the 23S rDNA. This Whole Genome Shotgun project has been deposited at DDBJ/ENA/GenBank under the accession PDIQ00000000. The version described in this paper is version PDIQ01000000. Genome sequences of *Streptomyces* sp. NP10 and related streptomycetes were analyzed for the presence of SM biosynthesis gene clusters using antiSMASH 4.02 (Blin et al., 2017a). The antiSMASH results were manually curated by performing multiple analyses of cluster-associated genes and their products.

### Generation of Recombinant Bacterial Strains, Plasmids, and General Growth Conditions

For genomic DNA isolation the *Streptomyces* strains were grown in 50 ml of 3% TSB medium (Oxoid, United Kingdom) in 250 ml baffled flasks at 28°C, 250 rpm. DNA was isolated using Wizard Genomic DNA Purification Kit (Promega, United States) as described before (Schaffert et al., 2016). All routine DNA standard techniques, cloning methods, and plasmid transformation into *Escherichia coli* were performed as described in Sambrook et al. (1989). PCR fragment amplifications were done with Q5^®^ High-Fidelity DNA Polymerase (New England Biolabs) using oligonucleotides listed in **Supplementary Table S1**. Plasmids and bacterial strains used or constructed during this study are listed in **Table 1**.

**Table 1:** List of plasmids and bacterial strains used in this study.

Plasmid	Description	Source
pSOK201	pSG5 minimal replicon, Am^R^, RP4 *ori*T, ColE1ori	Zotchev et al., 2000
pOSC12-2KN	pSG5 minimal replicon, Am^R^, RP4 *ori*T, ColE1ori, 550 bp of *fasB* from *Streptomyces* sp. NP10	This work
pKC1218H	pKC1218-derivate with SCP2^∗^ replicon; Hyg^R^	Sun et al., 2017
pOSC12-2EX	pKC1218H-derivate with SCP2^∗^ replicon; Hyg^R^; contains C12-2 BGC from *Streptomyces* sp. NP10	This work
pSOK806	ColE1ori, Am^R^, RP4oriT*,attP*_V WB,_*int*_V WB_, *ermE*^∗^p	Sekurova et al., 2016
*E. coli* XL1 Blue	general cloning host	New England Biolabs
*E. coli* ET12567/pUZ8002	strain for intergenic conjugation; Km^R^, Cm^R^	Flett et al., 1997
**Bacterial strain**		
**NP10^§^** (*Streptomyces* sp. NP10)	Wild-type (wt), ISS613, free fatty acids (FFAs) producer	Ilic-Tomic et al., 2015
**NP10_Apr** (*Streptomyces* sp. NP10/pSOK806)	wt strain harboring pSOK806(Am^R^)	This work
**NP10_Hyg** (*Streptomyces* sp. NP10/pKC1218H)	wt strain harboring pKC1218H(Hyg^R^)	This work
**mNP10** (*Streptomyces* sp. NP10/pOSC12-2KN)	C12-2 knockout mutant; wt strain with integrated pOSC12-2KN(Am^R^) for C12-2 disruption	This work
**mNP10_C12-2** (*Streptomyces* sp. NP10 C12-2KN/pOSC12-2EX)	complemented C12-2 knockout mutant; mNP10 harbouring pOSC12-2EX (Hyg^R^)	This work
*Streptomyces lividans* **TK24**	wt *Streptomyces lividans* TK24	Hopwood, 1999
**TK24_Hyg** (*S. lividans* TK24/pKC1218H)	*S. lividans* TK24wt strain harboring pKC1218H (Hyg^R^)	This work
**TK24_C12-2** (*S. lividans* TK24/pOSC12-2EX)	*S. lividans* TK24wt strain harboring pOSC12-2EX (Hyg^R^)	This work

*^§^Strain abbreviation (strain name).*

In order to inactivate the *fasB* gene, a *fasC-fasB* intergenic region of 550 bps was amplified with fasBkn_*Hind*III/ fasBkn_*EcoR*I primer pair from NP10 genomic DNA and cloned into the 3.1 kb *EcoR*I/*Hind*III fragment of the vector pSOK201 (**Table 1**). The generated plasmid pOSC12-2KN was transferred into the NP10 strain via conjugation and resulting *fasB*-disruption mutant mNP10 (NP10/pOSC12-2KN) was verified by PCR using fasB_fwd/fasC_rev primer pair.

To complement the inserted mutation in mNP10 (NP10/pOSC12-2KN) and to generate the recombinant strains for heterologous expression of cluster C12-2 in different *Streptomyces*, the pKC1218H-based plasmid pOSC12-2EX containing genes noted in **Table 5** was constructed. The DNA fragments encompassing genes from BGC C12-2 were amplified with primers given in **Supplementary Table S1**, generating three fragments, which were cloned into the pKC1218H through *Hind*III/*Spe*I/*EcoR*V/*EcoR*I restriction sites.

*Escherichia coli* strains were grown in Luria–Bertani (LB) broth or on LB agar, supplemented with chloramphenicol (25 μg ml^-1^), apramycin (100 μg ml^-1^), kanamycin (25 μg ml^-1^), and hygromycin (100 μg ml^-1^) as appropriate. XL1-blue was used for general cloning, ET12567 (pUZ8002) was used for intergeneric conjugative transfer of plasmids to *Streptomyces* as described before (Flett et al., 1997).

Spore suspensions of all *Streptomyces* strains were prepared in glycerol (20 %, v/v) (Kieser et al., 2000), maintained at -80°C, and used for the inoculation of cultures for further experiments. Starter culture was grown by inoculating spores (20 μl) into 100 ml flasks containing 20 ml vegetative medium (maltose 15 g l^-1^, tryptic soy broth 8 g l^-1^, yeast extract 4 g l^-1^, CaCO_3_ 2 g l^-1^) and incubated during 48 h in a shaking incubator set at 30°C and 180 rpm. These starter culture was used for the inoculation of different media (0.4%, v/v) in Erlenmeyer flasks containing coiled stainless steel wires for better aeration, and incubated in dark at 30°C and 180 rpm in shaking incubator. Mutant was also grown at 8°C.

### Free Fatty Acids Extraction and Analysis

For culture extract preparations *Streptomyces* strains were grown in 400 ml of MSY medium (maltose 30 g l^-1^, tryptic soy broth 8 g l^-1^, yeast extract 4 g l^-1^, CaCO_3_ 2 g l^-1^, NaNO_3_ 3 g l^-1^, MnSO_4_x7H_2_O 0.6 g l^-1^, ZnSO_4_ 0.005 g l^-1^, FeSO_4_x7H_2_O 0.3 g l^-1^, CoCl_2_x7H_2_O 5 mg l^-1^) complemented when needed with either apramycin (50 μg ml^-1^ for apramycin resistant constructs) or hygromycin (100 μg ml^-1^ for hygromycin resistant constructs) for 6 days at 30°C shaking at 180 rpm. Prior to extraction, 10% of the culture volume was taken out for dry cell mass (DCM) measurement. Total remaining cultures (mycelium and medium broth) were extracted with equal volume of hexane/chloroform mixture (4:1) for preparation of crude FFAs extracts. Organic phase was dried under reduced pressure and weighted.

The solvent (hexane:chloroform, 4:1, v/v) extracts of all *Streptomyces* strains listed in **Table 1**, were treated with an ethereal solution of CH_2_N_2_ in order to convert FFA to their corresponding methyl esters (FAMEs), thus allowing their quantitative and further qualitative analyses by GC-MS as described (Ilic-Tomic et al., 2015).

### Identification of Free Fatty Acids

The free FAs from NP10, mNP10, and mNP10_C12-2, were identified by GC-MS analysis as the corresponding methyl esters obtained after derivatization with CH_2_N_2_, as previously described (Ilic-Tomic et al., 2015). All analyzed total ion chromatograms contained several series of FAMEs showing regularities in their GC retention behavior (constant retention index difference of *ca.* 100 units) and possessing analogous mass spectra. The identification of saturated normal chain and branched (*iso*- and *anteiso*-) FAMEs was based on a combination of data coming from their mass spectra and gas chromatographic retention behavior. In addition to the mentioned spectral and retention data, the structure of monounsaturated normal and branched FAMEs, more specifically the double bond position, was inferred from the MS fragmentation patterns of the corresponding DMDS adducts. The identification was corroborated wherever possible by a subsequent GC-MS analysis of authentic standards. In order to clearly corroborate the presence of the cyclopropyl group in detected FAME, its identity was undoubtedly verified by co-injection of authentic sample obtained by cyclopropanation of methyl ester of 16:1ω7c using CH_2_N_2_ in the presence of Pd(PhCN)_2_Cl_2_ as the catalyst, whereas the position of branching methyl group in *i*-17:0cy9-10 (RI = 1966) was inferred from the corresponding ΔRI values. Data were presented as an average of at least two independent experiments.

## Results and Discussion

### General Features of the *Streptomyces* sp. NP10 Genome

**FIGURE 1:**
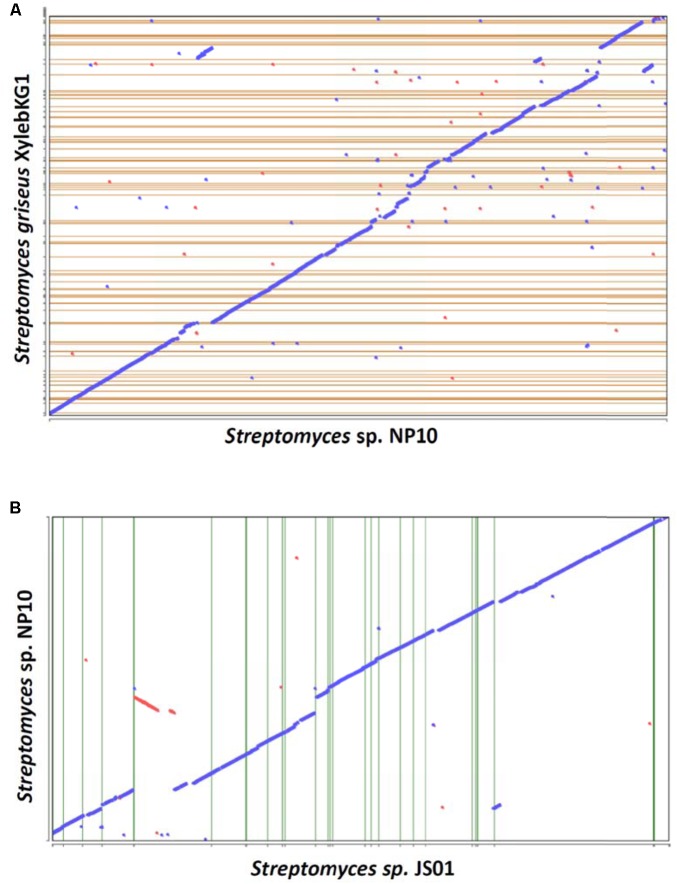
Genome synteny dotplots of *Streptomyces* sp. NP10 and closely related *Streptomyces griseus* XylebKG1 **(A)** and *Streptomyces* sp. JS01 **(B)**. The “blue” line represents the regions of similarities between the two genomes, and the discontinuities in this line represent regions of genomic variations at a given locus due to transposon insertions, deletions, or horizontal gene transfer. Red line and dots represent likely inversions.

A draft genome sequence of NP10 strain was obtained using Illumina MiSeq technology, yielding a genome of ca 7.7 Mb. The genome was annotated and some of the general data in comparison to the closely related *S. griseus* XylebKG-1 isolated from an ambrosia beetle (Grubbs et al., 2011), and *Streptomyces* sp. JS01 isolated from mangrove sediment and having inhibitory activity against algae *Phaeocystis globose* (Zhang et al., 2014) are given in **Table 2**. Using Joint Genomics Institute online tools, we compared the genomes of NP10 and XylebKG-1, and found them to have a high degree of synteny (**Figure 1A**). Considering this, and the high similarity of the housekeeping genes, it appears likely that strain NP10 belongs to the species *Streptomyces griseus*. Similarly, the synteny between NP10 and JS01 genomes was tested (**Figure 1B**), showing that the latter isolate is also closely related to *S. griseus* XylebKG-1 and NP10, but has larger genome rearrangements compared to the NP10. It is noteworthy that so far there have been no reports on high-level production of FAs by any *S. griseus* strain or *Streptomyces* sp. JS01.

**Table 2:** Comparison of genome data of *Streptomyces* sp. NP10, *S. griseus* XylebKG-1 and *Streptomyces* sp. JS01.

Genomic feature	*Streptomyces* sp. NP10	*S. griseus* XylebKG-1	*Streptomyces* sp. JS01
DNA, total number of bases	7,677,865	8,566,464	7,799,375
DNA G+C content, %	71.57	72.21	71.62
CRISPR count	2	1	8
Genes total number	6,935	7,450	7,106
Protein coding genes	6,851	7,379	7,019
rRNA operons	4	5	8
tRNA genes	65	66	71
Protein coding genes with function prediction	5,197	5,000	5,210
Protein coding genes connected to KEGG pathways	1,481	1,542	1,488
Protein coding genes with COGs	4,214	4,153	4,211
Genes in biosynthetic clusters	1,085	1,210	922
Protein coding genes coding signal peptides	463	664	482
Protein coding genes coding transmembrane proteins	1,684	1,720	1,727
Chromosomal cassettes	532	796	573

BLAST search of six housekeeping genes (*gap, glnA, gyrB, recA, rpoB*, and *rpoC*) revealed high degree of identity (>95% on nucleotide level) to the homologous genes in *S. griseus* XylebKG-1 confirming that isolate NP10 indeed belongs to the species *S. griseus*. Similar analysis was done for *Streptomyces* sp. JS01, whose six housekeeping genes were extracted from the JGI database and compared to their homologues from NP10. Even higher degree of identity (up to 99.9%) between the housekeeping genes from these strains was observed (**Supplementary Table S2**). Draft genome of NP10 was then subjected to the analysis by antiSMASH 4.02 (Blin et al., 2017a,b), the online platform for detection of gene clusters presumably involved in the biosynthesis of SMs. An extended search was performed, that included detection of putative gene clusters for FA biosynthesis. The results of the search were manually curated, eliminating some of the clusters that could not be clearly associated with secondary metabolism and splitting the others into separate clusters. Identical search and curation was performed for the genome of *S. griseus* XylebKG-1, and combined results are presented in **Table 3**. From the total of 34 SM BGC identified in this way in NP10 genome, 27 were also present in the genome of XylebKG-1. Interestingly, four gene clusters predicted to be involved in the biosynthesis of FAs were found in the NP10 genome, while only three were predicted to be present in XylebKG-1 genome. It seemed thus logical to assume, that the unique FA biosynthesis gene cluster designated C12-2 present in NP10 is responsible for the high-level production of FFAs by this strain. In an attempt to identify similar cluster in the genomes of other streptomycetes BLAST search was performed using C12-2 as a query. The one true hit of high-level identity (98–100% on individual gene level) was with the genome of *Streptomyces* sp. JS01.

**Table 3:** Secondary metabolite biosynthetic gene clusters (BGC) identified in the genome of *Streptomyces* sp. NP10 and their presence in the genomes of closely related species *S. griseus* XyelbKG-1 and *Streptomyces* sp. JS01 using antiSMASH 4.0 software followed by manual curation.

NP10 BGC No.	NP10 BGC type	Putative product	XyelbKG-1	JS01
2	Terpene	Terpenoid	+	+
8	Lantipeptide	AfmS-like class III lantipeptide	+	+
9	PKS I-NRPS	polyketide-NR peptide hybrid	+	+
12-1	Butyrolactone	γ-butyrolactone	+	+
**12-2**	**Fatty acid**	**fatty acids**	-	+
15	Lantipeptide	class II lantipeptides	+	+
16	Lasso peptide	Lasso peptide, class II	+	+
17	Ectoine-butyrolactone	unknown	-	-
18	Ectoine	ectoine	+	+
23	Fatty acid	fatty acids	+	+
27	Siderophore	desferrioxamine B	+	+
30	Siderophore	siderophore	+	+
34	Bacteriocin	bacteriocin	+	+
37	Fatty acid	fatty acids	+	+
42	PKS I-NRPS	polyketide-NR peptide hybrid	+	+
43	PKS III	alkylresorcinol	+	+
44	Melanin	melanin	+	+
45	Terpene	2-methyl isoborneol	+	+
46-1	Bacteriocin	linocin M18-like bacteriocin	+	+
46-2	PKS I-NRPS	polycyclic tetramate macrolactam	+	+
49	Terpene	lycopene-like terpenoid, glycosylated	-	-
55	PKS I-NRPS	diketopiperazines	-	+
56	NRPS	non-ribosomal peptide	-	-
61	Terpene	terpenoid	+	+
64	Fatty acid	fatty acids	+	+
68	Terpene	hopanoids	+	+
70-1	PKS III	1,3,6,8-tetrahydroxynaphthalene	+	+
70-2	Fatty acid	fatty acids	+	+
71	Terpene	isorenieratene	+	+
72	NRPS	griseobactin	+	+
73	PKS II-PKS I	polyketide, glycosylated	-	+
76	Terpene	geosmin	+	+
77	Butyrolactone	γ-butyrolactone	+	+
80	Terpene	terpenoid	-	-

*Gene cluster that has been subject to this study is in bold.*

antiSMASH 4.02 BGC prediction was performed for *Streptomyces* sp. JS01 in a manner similar to those done for *S. griseus* XylebKG-1 (**Table 3**) showing that JS01 shares even more identical BGCs with NP10 than XylebKG-1. In particular, NP10 and JS01 shared 30 of the 34 SM biosynthesis gene clusters, including C12-2.

### Characterization of the C12-2 Gene Cluster

**FIGURE 2:**
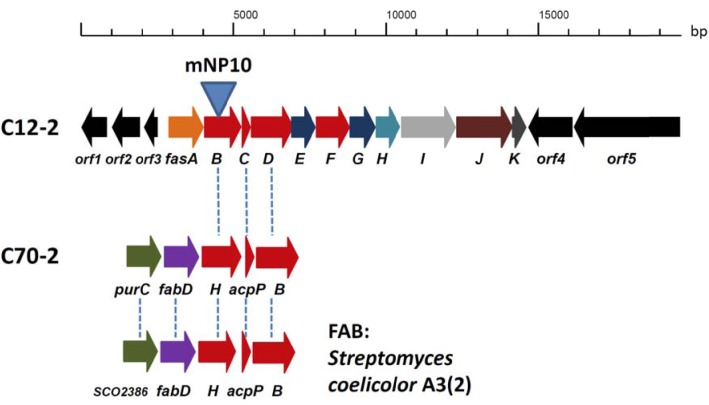
Organization of the C12-2 gene cluster presumably responsible for the overproduction of unusual fatty acids (FAs) in *Streptomyces* sp. NP10 (see **Table 5** for details on gene functions). Conventional FA biosynthesis sub-clusters featuring genes homologous to the ones present in C12-2, both from NP10 isolate (cluster C70-2) and *Streptomyces coelicolor* A3(2) are shown underneath. Homologous genes in the clusters are indicated with vertical dashed lines. Insertional inactivation of the cluster via disruption of *fasB* gene in the mNP10 mutant is indicated.

Genes involved in the biosynthesis of FAs in *Streptomyces* are normally organized in two sub-clusters (Gago et al., 2011). The first one includes genes coding for acetyl-CoA carboxylase (FabD - forms common extender unit malonyl-CoA via carboxylation of acetyl-CoA), 3-oxoacyl-ACP synthase III (Fab H – catalyzes the initial condensation of two acetate units), acyl carrier protein (AcpP), and 3-oxoacyl-ACP synthase II (FabB – catalyzes elongation of the FA chain). The second sub-cluster codes for the enzymes involved in the reductive steps of FA biosynthesis, ketoreductase FabG, dehydratase FabA, and enoyl reductase FabI. Based on the analyses described in the previous section, C12-2 was the only BGC that we could presume to be responsible for production of high amounts of various, non-canonical FFAs accumulated by *Streptomyces* sp. NP10. Gene cluster organization in comparison with the conventional FAS type II clusters from NP10 and *Streptomyces coelicolor* A3(2) are shown on **Figure 2**, and its genes and putative functions of their products are given in **Table 4**. Closest homologues of *orf1-3* were identified in *S. griseus* XylebKG1, while closest homologues of BGC *fasA-K* and downstream open reading frames *orf4-5* were identified in *Streptomyces* sp. JS01. In addition, *in silico* analysis of the C12-2 cluster ORFs revealed presence of a rare leucine codon TTA in the *fasI* ORF that is characteristic for SMs biosynthetic pathways controlled by the *bldA*-encoded tRNA (Hackl and Bechthold, 2015).

**Table 4:** Features of the BGC C12-2 presumed to be involved in the biosynthesis of unusual fatty acids (FAs) by *Streptomyces* sp. NP10.

Gene	Putative product	Size, aa	Putative function in FA biosynthesis
*orf1*	Hemolysin-type calcium-binding repeat protein	254	none
*orf2*	HAD-superfamily hydrolase	279	none
*orf3*	Hypothetical protein	139	none
*fasA*	A-factor biosynthesis hotdog domain protein	339	transfer of a β-keto acyl to the hydroxy group of DHAP
*fasB*	3-oxoacyl-ACP synthase III	364	initiation of the FA biosynthesis (FabH)
*fasC*	ACP	80	attachment of activated FAs (AcpP)
*fasD*	3-oxoacyl-ACP synthase II	412	condensation (elongation) steps in FA biosynthesis (FabB)
*fasE*	Enoyl-ACP reductase	237	reduction of a double bond in FA biosynthesis (FabG)
*fasF*	3-oxoacyl-ACP synthase III	327	initiation of the FA biosynthesis (FabH)
*fasG*	Enoyl- ACP reductase	247	reduction of a double bond in FA biosynthesis (FabG)
*fasH*	HAD-superfamily subfamily IB hydrolase	236	putative phosphatase
*fasI*	Major facilitator superfamily transporter	531	efflux of FAs
*fasJ*	FAD-dependent oxidoreductase	553	FA modification
*fasK*	Pyridoxamine 5′-phosphate oxidase-like enzyme	129	unknown
*orf4*	Arabinose efflux permease	446	none
*orf5*	AfsR-like transcriptional regulator	1061	none

*DHAP, dihydroxyacetone phosphate; FA, fatty acids.*

**FIGURE 3:**
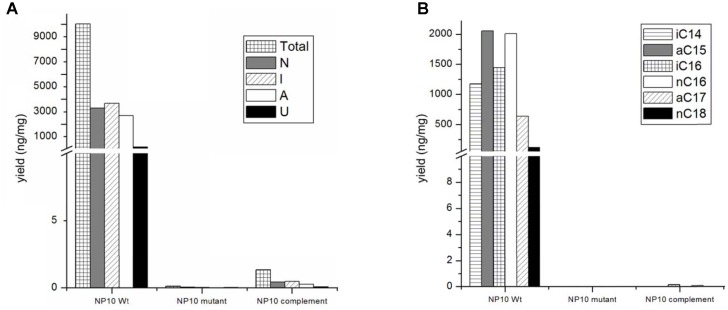
**(A)** Amounts of total, normal chained (N), *iso*-branched (I), *anteiso*-branched (A), and unsaturated (U) free fatty acids (FFAs), and **(B)** major classes of FFA produced by NP10 and their production in mNP10 and complemented mNP10 (mNP10_C12-2). Data is an average of two independent experiments.

**FIGURE 4:**
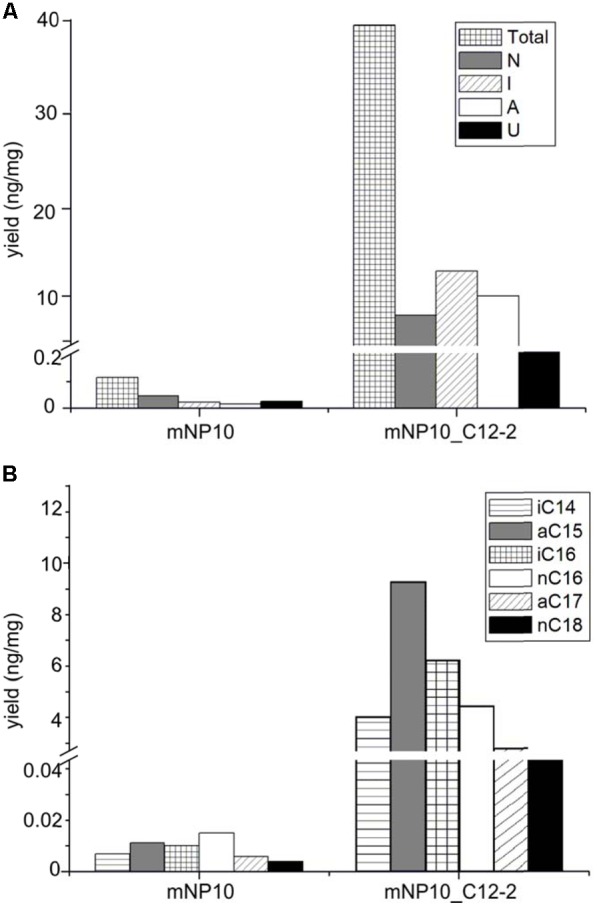
Comparison of FAA production in mNP10 and complemented strain mNP10_C12-2 by FA classes **(A)**, and regarding major FA products **(B)**. Data is an average of two independent experiments.

Judging from the genomic context, we assumed that the genes *fasA*-*fasK*, organized as an operon, might be responsible for the FFA biosynthesis. In order to verify this, intergenic region upstream of the *fasB* gene ORF encoding for 3-oxoacyl-ACP synthase II was amplified and cloned into the pSOK201-based suicide vector (Zotchev et al., 2000). The reason for choosing *fasB* for disruption was its presumed crucial role in the initiation steps in the FFA biosynthesis. The resulting plasmid pOSC12KN was introduced into NP10 by conjugation from *E. coli*, yielding several transconjugants. Three of these transconjugants were verified by PCR for correct integration of the construct into NP10 genome. All three were confirmed to be correct knock-out mutants, and one was chosen for further studies (**Supplementary Figure S1**). This mutant, designated mNP10 (**Table 1**), was subjected to fermentation along with the wild-type strain in the conditions typically resulting in high-level production of FFAs (Ilic-Tomic et al., 2015). The FFA content of the cultures and the results of the FFA identification analysis are presented in **Figures 3, 4** and **Table 5**. Evidently, disruption of *fasB* led to almost complete abrogation of FFA production in the mutant mNP10, supporting our hypothesis regarding the function of this gene cluster. The growth of this mutant was comparable, but somewhat delayed compared to the wild-type NP10 (data not shown). Contrary to the wild-type NP10, the mutant was not able to sporulate on solid media at 8°C, while in the liquid culture it produced 30% more biomass compared to NP10. Therefore, impaired accumulation of FFA appeared to be beneficial for the biomass formation, while influencing negatively morphological differentiation at lower temperatures. The latter effect can be related to the disturbance of membrane lipid homeostasis, which has been shown to affect differentiation in *S. coelicolor* (Arabolaza et al., 2010).

The gene content in the C12-2 cluster is unique with regard to putative roles of their products in the FA biosynthesis, and to our knowledge similar gene clusters have not been reported before. Homologues of FabH, encoded by *fasB* and *fasF*, are likely to be involved in the initiation of FFA biosynthesis. Despite proposed similar function, these proteins share only 20% identity, suggesting disparate roles, possibly in the utilization of the starter unit. The role of FasD would be to extend the starter units appended on the ACP encoded by *fasC*. Two putative enoyl-ACP reductases FasE and FasG, with predicted function of double bond reduction during FFA biosynthesis are quite dissimilar (only 36% identity), implying that they may have different substrate specificities. This seems plausible, considering structural diversity of FFAs produced by NP10 (Ilic-Tomic et al., 2015). Additional modification of FAs may be afforded through the action of enzymes encoded by *fasJ* (encodes putative FAD-dependent oxidoreductase) and *fasK* (encodes pyridoxamine 5′-phosphate-like enzyme), but the nature of such alterations is not possible to predict. Although the role of FasK in the biosynthesis of FFAs cannot be suggested at the moment, it is possible that FasJ has a function of ketoreductase or dehydratase. Alternatively, enzymes having the latter activities, such as FabG and FabA, as well as acetyl-CoA carboxylase FabD may be recruited from primary metabolism.

Enigmatic remains the role of AfsA homologue encoded by the *fasA* gene, which may be responsible for the generation of FA esters together with the putative phosphatase encoded by *fasH*, in analogy with the A-factor biosynthesis pathway (Kato et al., 2007). No such esters have been identified, however. Alternatively, a true butyrolactone, which has so far not been detected, may be biosynthesized by a concerted action of *fasA, fasH*, and *fasJ* gene products.

### Complementation of the mNP10 Mutant and Heterologous Expression of the C12-2 Cluster

The presumed complete C12-2 gene cluster was subsequently cloned into the pKC1218H vector, a modification of the low-copy autonomously replicating shuttle plasmid pKC1218 (Bierman et al., 1992) where the apramycin resistance marker was replaced with the hygromycin resistance gene (Sun et al., 2017). The cloning involved joining of three PCR-amplified parts of the C12-2 cluster, which were verified by DNA sequencing. The resulting plasmid designated pOSC12-2EX was introduced into the NP10 C12-2 mutant (mNP10), yielding recombinant strain mNP10_C12-2 (C12-2KN/pOSC12-2EX). Analysis of the FFA content of both strains revealed that insertional inactivation of the cluster resulted in almost complete abrogation of FFA accumulation in mNP10, while introduction of pOSC12-2EX into mNP10 restored FFA accumulation to ca 0.4% of the wild-type level (**Table 5** and **Figures 3, 4**). The total amounts of free FAs produced by strains mNP10, mNP10_C12-2, and wtNP10 were 0.12 ng mg^-1^, 40.3 ng mg^-1^, and 10021 ng mg^-1^ of dry cell weight, respectively (**Table 5**). Although the production of FFAs in mNP10 was almost completely abolished, different classes of FFAs were affected to a different extent. The production of unsaturated FFA was diminished ca 7 × 10^3^ fold, normal-chained FFAs were reduced ca 10^5^ fold, and levels of branched FFAs decreased over 1.6 × 10^5^ fold (**Table 5**).

**Table 5:** Total FFA profiles of the wild-type and the mutated strains of *Streptomyces* sp. NP10.

Compound	NP10 ng mg^-1a^	mNP10 ng mg^-1b^	mNP10_C12-2 ng mg^-1c^
Total	10021 (41)^d^	0.12 (28)	40.3 (24)
Saturated fatty acid methyl esters	9669 (29)	0.084 (20)	34.9 (16)
Normal chain (N)	3299 (18)	0.046 (11)	8.7 (7)
even-numbered	2895 (9)	0.038 (7)	6.5 (5)
odd-numbered	404 (9)	0.008 (4)	2.2 (2)
*Iso* (I)	3677 (7)	0.022 (6)	14.5 (6)
even-numbered	2712 (4)	0.017 (3)	10.8 (3)
odd-numbered	965 (3)	0.005 (3)	3.7 (3)
*Anteiso* (A)	2693 (4)	0.016 (3)	11.7 (3)
even-numbered	n.d.^e^	n.d.	n.d.
odd-numbered	2693 (4)	0.016 (3)	11.7 (3)
Unsaturated fatty acid methyl esters (U)	175 (7)	0.025 (6)	3.3 (4)
Normal chain	175 (4)	0.025 (6)	2.3 (2)
*Iso*	tr^f^ (2)	n.d.	tr (1)
*Anteiso*	tr (1)	n.d.	1.0 (1)
3-Hydroxy fatty acid methyl esters (H)	tr (1)	n.d.	n.d.
Cyclopropane fatty acid methyl esters (CP)	177 (4)	0.005 (2)	2.1 (4)

*^a,b,c^Concentration is expressed as ng per mg of the dry mycelium of (a) NP10, (b) mNP10, and (c) mNP10_C12-2; ^d^the number in brackets represents the number of compounds belonging to that class; ^e^n.d., not detected; ^f^tr - trace (<0.001 ng mg^-1^).*

Although low-level complementation is not uncommon in *Streptomyces* (Nedal et al., 2007), and may depend on whether and where the complementing genes are located, we decided to test a possibility of heterologous expression of the C12-2 gene cluster. For this purpose, *Streptomyces lividans* TK24, often used for heterologous gene expression was chosen. Plasmid pOSC12-2EX was introduced into *S. lividans* TK24 and production of FFAs by the recombinant strain TK24_C12-2 (**Table 1**) was investigated as compared with the controls (TK24wt and TK24_Hyg, strain carrying empty vector pKC1218H). No high-level production of FFAs was detected by any of these strains (**Supplementary Figure S2**), suggesting that the cloned gene cluster may be not expressed in *S. lividans*, or some other genes, possibly involved in regulation of precursor supply in NP10 are needed for proper functioning of this gene cluster. Another possibility is that the presence of TTA codon in the *fasI* gene hampers efficient translation, and thus impairs functional expression of the cluster. We have also observed very low segregational stability of the pKC1218H vector even in the presence of hygromycin. Most likely, hygromycin phosphotransferase encoded by the vector is efficiently expressed and inactivates antibiotic early during the growth, thereby eliminating selection pressure needed to maintain the vector during the fermentation.

### Identification of FFAs Produced by the Recombinant Strains

Detailed characterization of the FFAs produced by mNP10 and C12-2-complemented mNP10 strains with respect to total FFA production per mg of dry mass mycelium, amount of normal-chain, branched and unsaturated FFAs as well as the amount of most abundant FFAs is presented on **Figures 3A,B**. Complete profile of FFAs produced by mNP10 and mNP10(C12-2) in comparison with the wild-type strain NP10 is presented in **Table 5**, and detailed analysis of individual FAs is given in **Supplementary Table S3**. Notably, not only the total amount of FFAs decreased drastically, but also the diversity of most abundant FFAs decreased from 40 different acids detected in the wild-type strain to 28 detected in mNP10 (**Supplementary Table S3**). Diversity of normal-chained FFAs was the most affected, while branched and unsaturated FFA diversity was less influenced in the knock-out mutant.

Comparison of mNP10 and complemented strain mNP10_C12-2 showed partial restoration of biosynthesis of all classes of FFAs (**Figure 4**), but the production of some of the saturated medium C-chain length (C8-12) FFAs, both normal and branched, failed to be restored even in traces (**Supplementary Table S3**). The largest contributors to the total FFA content of the mNP10(C12-2) were the saturated FAs, mainly the branched ones, with long *iso*- and *anteiso*- chain being predominant, with only a minor portion of unsaturated FA and cyclopropane FA detected (**Table 5**). When compared to mNP10, the increase of FFA levels in the complemented strain mNP10(C12-2) was approximately 700-fold for total branched-chain FFAs, and approximately 400-fold for normal-chain FFAs. Free FA profile of mNP10_C12-2 was dominated by saturated *anteiso*-FA with *a*-15:0 (9.26 ng mg^-1^), *a*-17:0 (2.47 ng ml^-1^), and *iso*-FA with *i*-16:0 (6.16 ng mg^-1^) as the most abundant ones. These branched chain FAs were followed by normal chain isomer 16:0 (4.26 ng mg^-1^). Interestingly, while the wild-type strain and the knock-out mutant produced only traces of some FA methyl esters, in particular methyl(Z)-9-octadecenoate, methyl-8-(2-(4-methylpentyl)cyclopropyl-octanoate, and methyl(Z)-14-methylhexadec-9-enoate, complemented mutant produced measurable amounts of these FAs. It seems likely, therefore, that enzymes encoded by the C12-2 gene cluster are particularly important for the production of such metabolites.

## Conclusion

Unprecedented ability of *Streptomyces* sp. NP10 to produce copious amounts of structurally diverse FAs (Ilic-Tomic et al., 2015) prompted us to gain a genome-based insight into this phenomenon. Although genome of NP10 proved to be very similar to those of several strains belonging to *S. griseus* clade, comparative analysis aimed at identification of possible FA synthases helped to identify a candidate gene cluster. Subsequent gene inactivation and complementation proved this cluster to be involved in the biosynthesis of various FFAs in NP10. The cluster appears to be unique, since such gene combination has never been reported for a FA biosynthesis gene cluster. This study provides a platform for detailed analyses of these genes and their products, which will allow identification for their roles in the biosynthesis of structurally diverse FFAs.

## Author Contributions

JN-R, BV, NR, and SZ conceived the project, conducted data analysis, and wrote the manuscript. OS performed all genetic manipulations. TI-T and NS performed fermentations, extractions, purifications. MG, MŽ, and NR performed FFA identification and quantification. CR and JK sequenced, assembled, and analyzed the genome. All authors reviewed and discussed the manuscript, and contributed to its writing.

## Conflict of Interest Statement

The authors declare that the research was conducted in the absence of any commercial or financial relationships that could be construed as a potential conflict of interest.

## References

[B1] Arabolaza A. D’Angelo M. Comba S. Gramajo H. (2010). FasR, a novel class of transcriptional regulator, governs the activation of fatty acid biosynthesis genes in Streptomyces coelicolor. *Mol. Microbiol.* 78 47–63. 10.1111/j.1574-6976.2010.00259.x 20624224

[B2] Baltz R. H. (2017). Gifted microbes for genome mining and natural product discovery. *J. Ind. Microbiol. Biotechnol.* 44 573–588. 10.1007/s10295-016-1815-x 27520548

[B3] Bierman M. Logan R. O’Brien K. Seno E. T. Rao R. N. Schoner B. E. (1992). Plasmid cloning vectors for the conjugal transfer of DNA from *Escherichia coli* to *Streptomyces* spp. *Gene* 116 43–49. 10.1016/0378-1119(92)90627-2 1628843

[B4] Blin K. Medema M. H. Kottmann R. Lee S. Y. Weber T. (2017a). The antiSMASH database, a comprehensive database of microbial secondary metabolite biosynthetic gene clusters. *Nucleic Acids Res.* 45 D555–D559. 10.1093/nar/gkw960 27924032 PMC5210647

[B5] Blin K. Wolf T. Chevrette M. G. Lu X. Schwalen C. J. Kautsar S. A. (2017b). AntiSMASH 4.0-improvements in chemistry prediction and gene cluster boundary identification. *Nucleic Acids Res.* 45 W36–W41. 10.1093/nar/gkx319 28460038 PMC5570095

[B6] Chater K. F. (2016). Recent advances in understanding *Streptomyces*. *F1000Res.* 5:2795. 10.12688/f1000research.9534.1 27990276 PMC5133688

[B7] Flett F. Mersinias V. Smith C. P. (1997). High efficiency intergeneric conjugal transfer of plasmid DNA from *Escherichia coli* to methyl DNA-restricting streptomycetes. *FEMS Microbiol. Lett.* 155 223–229. 10.1111/j.1574-6968.1997.tb13882.x 9351205

[B8] Gago G. Diacovich L. Arabolaza A. Tsai S. C. Gramajo H. (2011). Fatty acid biosynthesis in actinomycetes. *FEMS Microbiol. Rev.* 35 475–497. 10.1111/j.1574-6976.2010.00259.x 21204864 PMC3079561

[B9] Gordon D. Green P. (2013). Consed: a graphical editor for next-generation sequencing. *Bioinformatics* 29 2936–2937. 10.1093/bioinformatics/btt515 23995391 PMC3810858

[B10] Grubbs K. J. Biedermann P. H. Suen G. Adams S. M. Moeller J. A. Klassen J. L. (2011). Genome sequence of *Streptomyces griseus* strain XylebKG-1, an ambrosia beetle-associated actinomycete. *J. Bacteriol.* 193 2890–2891. 10.1128/JB.00330-11 21460079 PMC3133108

[B11] Hackl S. Bechthold A. (2015). The gene *bldA*, a regulator of morphological differentiation and antibiotic production in *Streptomyces*. *Arch. Pharm.* 348 455–462. 10.1002/ardp.201500073 25917027

[B12] Han L. Lobo S. Reynolds K. A. (1998). Characterization of betaketoacyl-acyl carrier protein synthase III from *Streptomyces glaucescens* and its role in initiation of fatty acid biosynthesis. *J. Bacteriol.* 180 4481–4486. 9721286 10.1128/jb.180.17.4481-4486.1998 PMC107458

[B13] Handke P. Lynch S. A. Gill R. T. (2011). Application and engineering of fatty acid biosynthesis in *Escherichia coli* for advanced fuels and chemicals. *Metab. Eng.* 13 28–37. 10.1016/j.ymben.2010.10.007 21056114

[B14] Hopwood D. A. (1999). Forty years of genetics with Streptomyces: from in vivo through in vitro to in silico. *Microbiology* 145 2183–2202. 10.1099/00221287-145-9-2183 10517572

[B15] Ilic-Tomic T. Genčić M. S. Živković M. Z. Vasiljevic B. Djokic L. Nikodinovic-Runic J. (2015). Structural diversity and possible functional roles of free fatty acids of the novel soil isolate *Streptomyces* sp. NP10. *Appl. Microbiol. Biotechnol.* 99 4815–4833. 10.1007/s00253-014-6364-5 25636833

[B16] Kato J. Y. Funa N. Watanabe H. Ohnishi Y. Horinouchi S. (2007). Biosynthesis of gamma-butyrolactone autoregulators that switch on secondary metabolism and morphological development in *Streptomyces*. *Proc. Natl. Acad. Sci. U.S.A.* 104 2378–2383. 10.1073/pnas.0607472104 17277085 PMC1892969

[B17] Kealey C. Creaven C. A. Murphy C. D. Brady C. B. (2017). New approaches to antibiotic discovery. *Biotechnol. Lett.* 39 805–817. 10.1007/s10529-017-2311-8 28275884

[B18] Kieser T. Bibb M. J. Buttner M. J. Chater K. F. Hopwood D. A. (2000). *Practical Streptomyces Genetics.* Norwich: John Innes Centre; Norwich Research Park.

[B19] Nedal A. Sletta H. Brautaset T. Borgos S. E. Sekurova O. N. Ellingsen T. E. (2007). Analysis of the mycosamine biosynthesis and attachment genes in the nystatin biosynthetic gene cluster of *Streptomyces noursei* ATCC 11455. *Appl. Environ. Microbiol.* 73 7400–7407. 10.1128/AEM.01122-07 17905880 PMC2168226

[B20] Revill W. P. Bibb M. J. Scheu A. K. Kieser H. J. Hopwood D. A. (2001). Beta-ketoacyl acyl carrier protein synthase III (FabH) is essential for fatty acid biosynthesis in *Streptomyces coelicolor* A3(2). *J. Bacteriol.* 183 3526–3530. 10.1128/JB.183.11.3526-3530.2001 11344162 PMC99652

[B21] Sambrook J. Fritsch E. F. Maniatis T. (1989). *Molecular Cloning: A Laboratory Manual.* Cold Spring Harbor, NY: Cold Spring Harbor Laboratory.

[B22] Schaffert L. Albersmeier A. Winkler A. Kalinowski J. Zotchev S. B. Rückert C. (2016). Complete genome sequence of the actinomycete *Actinoalloteichus hymeniacidonis* type strain HPA 177^T^ isolated from a marine sponge. *Stand. Genomic Sci.* 11:91. 10.1186/s40793-016-0213-3 28031775 PMC5168871

[B23] Seemann T. (2014). Prokka: rapid prokaryotic genome annotation. *Bioinformatics* 30 2068–2069. 10.1093/bioinformatics/btu153 24642063

[B24] Sekurova O. N. Zhang J. Kristiansen K. A. Zotchev S. B. (2016). Activation of chloramphenicol biosynthesis in *Streptomyces venezuelae* ATCC 10712 by ethanol shock: insights from the promoter fusion studies. *Microb. Cell Fact.* 15:85. 10.1186/s12934-016-0484-9 27206520 PMC4875748

[B25] Singh R. Reynolds K. A. (2016). Identification and characterization of FabA from the type II fatty acid synthase of *Streptomyces coelicolor*. *J. Nat. Prod.* 79 240–243. 10.1021/acs.jnatprod.5b00560 26731437

[B26] Sun Y. Q. Busche T. Rückert C. Paulus C. Rebets Y. Novakova R. (2017). Development of a biosensor concept to detect the production of cluster-specific secondary metabolites. *ACS Synth. Biol.* 16 1026–1033. 10.1021/acssynbio.6b00353 28221784

[B27] van der Meij A. Worsley S. F. Hutchings M. I. van Wezel G. P. (2017). Chemical ecology of antibiotic production by actinomycetes. *FEMS Microbiol. Rev.* 41 392–416. 10.1093/femsre/fux005 28521336

[B28] Zhang H. Zhang S. Peng Y. Li Y. Chen Z. Zheng W. (2014). Draft genome sequence of the anti-algal marine actinomycete Streptomyces sp. JS01. *Genome Announc.* 2:e1261-14. 10.1128/genomeA.01261-14 25477414 PMC4256195

[B29] Zheng C. J. Sohn M. J. Chi S. W. Kim W. G. (2010). Methyl-branched fatty acids, inhibitors of enoyl-ACP reductase with antibacterial activity from *Streptomyces* sp. A251. *J. Microbiol. Biotechnol.* 20 875–880. 10.4014/jmb.1001.01004 20519910

[B30] Zotchev S. Haugan K. Sekurova O. Sletta H. Ellingsen T. E. Valla S. (2000). Identification of a gene cluster for antibacterial polyketide-derived antibiotic biosynthesis in the nystatin producer *Streptomyces noursei* ATCC 11455. *Microbiology* 146 611–619. 10.1099/00221287-146-3-611 10746764

