# Supplementary material for: Genomics-Based Insights Into the Biosynthesis and Unusually High Accumulation of Free Fatty Acids by Streptomyces sp. NP10
Source: Front Microbiol. 2018 Jun 19;9:1302. doi: 10.3389/fmicb.2018.01302 (PMC6018390; doi:10.3389/fmicb.2018.01302)

**Supplementary Materials:**

**Genomics-based insights into the biosynthesis and unusually high accumulation of free fatty acids by *Streptomyces* sp. NP10**

Olha Schneider, Tatjana Ilic-Tomic, Christian Rückert, Jörn Kalinowski, Marija S. Genčić, Milena Z. Živković, Nada Stankovic, Niko S. Radulović, Branka Vasiljevic, Jasmina Nikodinovic-Runic, Sergey B. Zotchev

**Table S1.** List of oligonucleotides used in this study (Italic: endonuclease restriction enzyme site)

| Oligonucleotide | Sequence (5′ − 3′) |
| --- | --- |
| fasBkn_*Hind*III | CGATAAG*AAGCTT*ACCACGCGGTGCCGATCGGACTGC |
| fasBkn_*EcoR*I | GAAAGC*AATTC*GCGACGGACTCCGTCAATGG |
| fasB_fwd | GTTCGGACGCTTCGTCGAACTG |
| fasC_rev | TCTCCACGGCGACGCTGATCTC |
| fasBex1_*Hind*III | CGATAAG*AAGCTT*TGGCGGATGCTCCCTCGATGG |
| fasBex1_*EcoR*V | CGCCGACT*GATATC*ACGCCGATTGCCATGGATT |
| fasBex2_*EcoR*V | ATCCATGGCAATCGGCGT*GATATC*AGTCGG |
| fasBex2_*Spe*I | CCTTGTGTAAGG*ACTAGT*TTATATC |
| fasBex3_*Spe*I | GATATAA*ACTAGT*GTCCTTACACAAGGAATC |
| fasBex3_*EcoR*I | GAAAGC*GAATTC*TTCGGGCCACGGCCCATGCTGCTGGT |

**Table S2.** Comparative analysis of six housekeeping genes in *Streptomyces* spp. NP10 and JS01.

| **Annotation** | **NP10 vs. JS01 identity, %** | |
| --- | --- | --- |
|  | **Gene** | **Protein** |
| DNA-directed RNA polymerase, beta subunit, *rpoB* | 99.5 | 99.9^a^ |
| Glycerol kinase, *glpK* | 99.1 | 99.6 |
| ATP synthase F1, β-subunit, *atpD* | 97.9 | 97.9 |
| DNA gyrase, β-subunit, *gyrB* | 98.6 | 99.1 |
| RecA recombinase, *recA* | 99.0 | 99.2 |
| Tryptophan synthase, β-subunit, *trpB* | 98.9 | 99.5 |

^a^1 amino acid substitution

**Table S3.** Detailed free fatty acid profiles of the wild-type and the mutated strains of *Streptomyces* sp. NP10.

| **RI^a^** | **Compound** | **Designation** | **Class** | **NP10**  **ng mg^-1b^** | **mNP10**  **ng mg^-1c^** | **mNP10_C12-2**  **ng mg^-1d^** | **Method of identification^e^** |
| --- | --- | --- | --- | --- | --- | --- | --- |
| 1022 | Methyl heptanoate | 7:0 | N | tr^f^ | n.d^g^ | n.d. | RI, MS, CoI |
| 1122 | Methyl octanoate | 8:0 | N | 39.5 | 0.004 | n.d. | RI, MS, CoI |
| 1222 | Methyl nonanoate | 9:0 | N | 90.2 | tr | n.d. | RI, MS, CoI |
| 1298 | Methyl (*Z*)-4-decenoate | 10:1ω6c | U | n.d. | 0.004 | n.d. | RI, MS |
| 1255 | Methyl 3-hydroxyoctanoate | 3-OH-8:0 | H | tr | n.d. | n.d. | RI, MS |
| 1287 | Methyl 8-methylnonanoate | *i*-10:0 | I | tr | n.d. | n.d. | RI, MS |
| 1323 | Methyl decanoate | 10:0 | N | tr | tr | n.d. | RI, MS, CoI |
| 1374 | Methyl 2-decenoate^m^ | 10:1ω8 | U | n.d. | 0.004 | n.d. | RI, MS |
| 1396 | Methyl 8-methyldecanoate | *a*-11:0 | A | tr | n.d. | n.d. | RI, MS |
| 1413 | Methyl 10-undecenoate | 11:1ω1 | U | tr | n.d. | n.d. | RI, MS, CoI |
| 1424 | Methyl undecanoate | 11:0 | N | tr | n.d. | n.d. | RI, MS, CoI |
| 1488 | Methyl 10-methylundecanoate | *i*-12:0 | I | n.d. | n.d. | n.d. | RI, MS |
| 1499 | Methyl (*Z*)-9-dodecenoate | 12:1ω3c | U | n.d. | 0.005 | n.d. | RI, MS |
| 1524 | Methyl dodecanoate | 12:0 | N | n.d. | 0.005 | n.d. | RI, MS, CoI |
| 1588 | Methyl 11-methyldodecanoate | *i*-13:0 | I | 65.7 | tr | tr | RI, MS |
| 1596 | Methyl 10-methyldodecanoate | *a*-13:0 | A | tr | tr | tr | RI, MS |
| 1624 | Methyl tridecanoate | 13:0 | N | 36.2 | tr | n.d. | RI, MS, CoI |
| 1688 | Methyl 12-methyltridecanoate | *i*-14:0 | I | 1174 | 0.007 | 3.79 | RI, MS |
| 1696 | Methyl 11-methyltridecanoate | *a*-14:0 | A | n.d. | n.d. | n.d. | RI, MS |
| 1699 | Methyl (*Z*)-9-tetradecenoate | 14:1ω5c | U | n.d. | 0.007 | n.d. | RI, MS |
| 1724 | Methyl tetradecanoate | 14:0 | N | 287 | 0.007 | 0.98 | RI, MS, CoI |
| 1788 | Methyl 13-methyltetradecanoate | *i*-15:0 | I | 586 | 0.005 | 2.39 | RI, MS |
| 1796 | Methyl 12-methyltetradecanoate | *a*-15:0 | A | 2057 | 0.011 | 9.26 | RI, MS |
| 1824 | Methyl pentadecanoate | 15:0 | N | 164 | 0.004 | 1.14 | RI, MS, CoI |
| 1863 | Methyl (*Z*)-14-methylpentadec-9-enoate | *i*-16:1ω6c | U | tr | n.d. | n.d. | RI, MS, DMDS |
| 1888 | Methyl 14-methylpentadecanoate | *i*-16:0 | I | 1449 | 0.010 | 6.15 | RI, MS, CoI |
| 1896 | Methyl 13-methylpentadecanoate | *a*-16:0 | A | n.d. | n.d. | n.d. | RI, MS |
| 1899 | Methyl (*Z*)-9-hexadecenoate | 16:1ω7c | U | 175 | 0.005 | 1.03 | RI, MS, CoI, DMDS |
| 1924 | Methyl hexadecanoate | 16:0 | N | 2006 | 0.015 | 4.26 | RI, MS, CoI |
| 1966 | Methyl 8-(2-(4-methylpentyl)cyclopropyl)octanoate | *i*-17:0cy9-10 | CP | tr | tr | 0.94 | RI, MS |
| 1971 | Methyl (*Z*)-14-methylhexadec-9-enoate | *a*-17:1ω7c | U | tr | n.d. | 1.05 | RI, MS, DMDS |
| 1988 | Methyl 15-methylhexadecanoate | *i*-17:0 | I | 314 | tr | 1.34 | RI, MS |
| 1996 | Methyl 14-methylhexadecanoate | *a*-17:0 | A | 636 | 0.006 | 2.47 | RI, MS |
| 1999 | Methyl (*Z*)-9-heptadecenoate | 17:1ω8c | U | tr | n.d. | n.d. | RI, MS, DMDS |
| 2002 | Methyl 8-(2-hexylcyclopropyl)octanoate | 17:0cy9-10 | CP | 177 | 0.005 | 1.12 | RI, MS, CoI |
| 2024 | Methyl heptadecanoate | 17:0 | N | 114 | 0.004 | 1.02 | RI, MS, CoI |
| 2063 | Methyl (*Z*)-16-methylheptadec-9-enoate | *i*-18:1ω8c | U | tr | n.d. | tr | RI, MS, DMDS |
| 2074 | Methyl 8-(2-(4-methylhexyl)cyclopropyl)octanoate | *a*-18:0cy9-10 | CP | tr | n.d. | tr | RI, MS |
| 2088 | Methyl 16-methylheptadecanoate | *i*-18:0 | I | 89 | tr | 0.86 | RI, MS |
| 2099 | Methyl (*Z*)-9-octadecenoate | 18:1ω9c | U | tr | tr | 1.27 | RI, MS, CoI, DMDS |
| 2124 | Methyl octadecanoate | 18:0 | N | 563 | 0.007 | 1.27 | RI, MS, CoI |
| 2202 | Methyl 8-(2-octylcyclopropyl)octanoate | 19:0cy9-10 | CP | tr | n.d. | tr | RI, MS |
| 2224 | Methyl nonadecanoate | 19:0 | N | tr | n.d. | n.d. | RI, MS, CoI |
| 2324 | Methyl icosanoate | 20:0 | N | tr | tr | tr | RI, MS, CoI |
| 2524 | Methyl docosanoate | 22:0 | N | tr | n.d. | tr | RI, MS, CoI |
| 2625 | Methyl tricosanoate | 23:0 | N | tr | n.d. | n.d. | RI, MS |
| 2926 | Methyl hexacosanoate | 26:0 | N | tr | n.d. | n.d. | RI, MS |
| 3026 | Methyl heptacosanoate | 27:0 | N | tr | n.d. | n.d. | RI, MS |
| 3126 | Methyl octacosanoate | 28:0 | N | tr | n.d. | n.d. | RI, MS |

^a^ RI − Retention indices on a DB-5 column calculated against a series of co-injected *n*-alkanes (C_6_‒C_34_);

^b,c,d^ concentration is expressed as ng per mg of the dry mycelium of (a) NP10, (b) mNP10, and (c) mNP10_C12-2;

^e^ RI − Constituent identified by retention index matching; MS − Constituent identified by mass spectra comparison; CoI − The identity of the constituent was additionally confirmed by co-injection of an authentic sample; DMDS ‒ Position of double bond was confirmed by formation of corresponding dimethyl disulphide adducts;

^f^ tr − trace (<0.001 ng mg^-1^);

^g^ n.d. − not detected.

**Figure S1.**  *fasB* gene disruption in the genome of *Streptomyces* sp. NP10. (A) Integration of the gene disruption vector into the *fasB* gene; (B) C12-2 chromosomal locus of mNP10; (C) Analytical PCR of NP10 and mNP10 genomes using primers fasB_fwd and fasC_rev (Table S1): 1- control, product from NP10 as template (1415 bps); 2- GeneRuler 1 kb DNA Ladder (ThermoFisher Scientific); 3 - PCR product from genomic DNA isolated from one of the knock-out mutants mNP10_C12-2 as template (5084 bps).

C

**Figure S2.** (A) Amounts of total (T), normal chained (N), *iso*-branched (I), *anteiso*-branched (A), unsaturated (U) and cyclopropyl free fatty acids (CP), and (B) major classes of FFA produced by *S. lividans* TK21 and their production in *S. lividans* TK21_Hyg and heterolougously expressing C12-2 (*S. lividans* TK21_C12-2). Data is an average of two independent experiments.


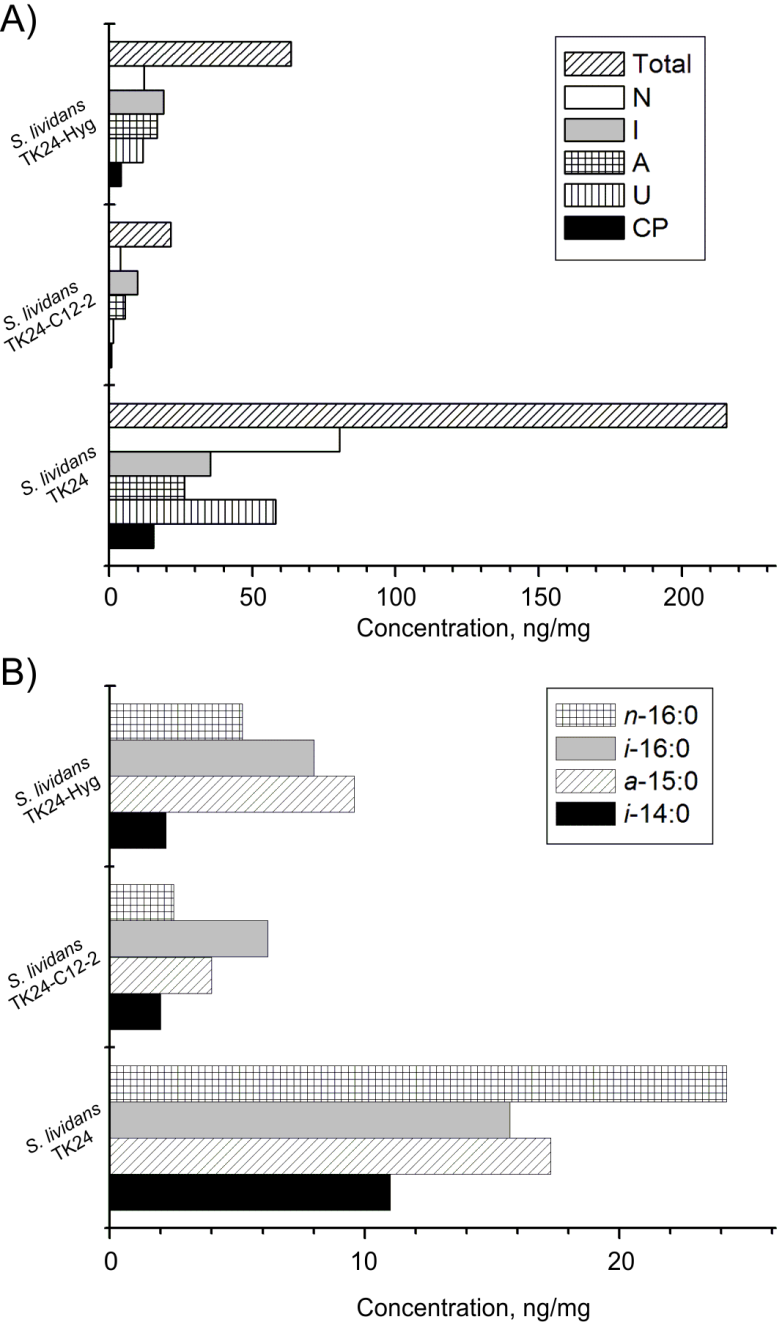

Supplement: Supplementary file 1 [file Data_Sheet_1.docx]
